# Effects of Aflatoxin B_1_ and Fumonisin B_1_ on Blood Biochemical Parameters in Broilers

**DOI:** 10.3390/toxins2040453

**Published:** 2010-03-29

**Authors:** Eliana N. C. Tessari, Estela Kobashigawa, Ana Lúcia S. P. Cardoso, David R. Ledoux, George E. Rottinghaus, Carlos A. F. Oliveira

**Affiliations:** 1 Center for Advanced Technological Research on Poultry, Biological Institute, Descalvado, SP, Brazil; Email: etessari@biologico.sp.gov.br (E.N.C.T.); alspcardoso@biologico.sp.gov.br (A.L.S.P.C.); 2 Department of Food Engineering, School of Animal Science and Food Engineering, University of São Paulo, Pirassununga, SP, Brazil; Email: ekoba@usp.br (E.K.); 3 Department of Animal Science, University of Missouri, Columbia, Missouri, USA; Email: LedouxD@missouri.edu (D.R.L.); 4 Veterinary Medical Diagnostic Laboratory, College Veterinary Medicine, University of Missouri, Columbia, Missouri, USA; Email: RottinghausG@missouri.edu (G.E.R.)

**Keywords:** AFB_1_, FB_1_, broiler chickens, toxic effects

## Abstract

The individual and combined effects of dietary aflatoxin B_1 _(AFB_1_) and fumonisin B_1_ (FB_1_) on liver pathology, serum levels of aspartate amino-transferase (AST) and plasma total protein (TP) of broilers were evaluated from 8 to 41 days of age. Dietary treatments included a 3 × 3 factorial arrangement with three levels of AFB_1 _(0, 50 and 200 μg AFB_1_/kg), and three levels of FB_1 _(0, 50 and 200 mg FB_1_/kg). At 33 days post feeding, with the exception of birds fed 50 mg FB_1 _only, concentrations of AST were higher (p < 0.05) in all other treatment groups when compared with controls. Plasma TP was lower (p < 0.05) at six days post feeding in groups fed 200 μg AFB_1_/kg alone or in combination with FB_1_. At day 33 days post feeding, with the exception of birds fed the highest combination of AFB_1 _and FB_1 _which had higher plasma TP than control birds_, _plasma TP of birds fed other dietary treatments were similar to controls. Broilers receiving the highest levels of AFB_1_ and FB_1_ had bile duct proliferation and trabecular disorder in liver samples. AFB_1_ singly or in combination with FB at the levels studied, caused liver damage and an increase in serum levels of AST.

## 1. Introduction

Mycotoxins are secondary toxic metabolites that are produced by fungi growing on food products, such as corn, peanut, and wheat, among others [1]. Exposure occurs predominantly by the ingestion of contaminated feed, when contaminated cereals such as corn, wheat, peanuts and sorghum, as well as other raw materials, are used in the preparation of animal feed [2].

Aflatoxins are produced by fungi of the genus *Aspergillus,* particularly *A. flavus, A. parasiticus* and *A. nomius* [3]. Seventeen metabolites have been identified as aflatoxins, with aflatoxin B_1_ (AFB_1_) being the most commonly found metabolite in cereals and the one that exhibits the highest toxigenic effects [4]. Biochemically, aflatoxins affect energy, carbohydrates and lipids, nucleic acids and protein metabolism [5]. Their biological effects include carcinogenicity, mutagenicity, teratogenicity and hepatotoxicity [6]. Aflatoxins are a frequent problem for poultry production resulting in poor bird performance [4], which is caused by several factors including reduced activity of pancreatic enzymes, decreased concentration of bile [6], increased incidence of leg problems, injury to the sciatic nerve [7], and antagonism in the metabolism of vitamins, proteins and amino acids, lipids and carbohydrates, and damage to DNA [6,8,9]. One of the most important effects of this toxin is the inhibition of protein synthesis, causing a marked reduction in the level of plasma protein, mainly α and β globulins, and albumin [10]. Also, the activity of serum or plasma enzymes such as aspartate amino-transferase (AST) has been extensively used as a measure of aflatoxin toxicity in chickens [7]. 

Fumonisins are secondary toxic metabolites produced by fungi belonging to the genus *Fusarium*, mainly the species *Fusarium verticillioides* [11]. To date, 16 fumonisins have been identified [1], however the predominant toxin produced by *F. verticillioides* strains is fumonisin B_1_ (FB_1_). Fumonisin B_1_ is the most abundant and toxic of the fumonisins, representing about 70% of the total contamination of food and feed naturally contaminated [12].

Pigs and horses are more susceptible to the toxic effects of fumonisin than most domestic birds [4]. In broilers, more severe symptoms such as diarrhea, decreased feed consumption, decreased body weight gain, increased relative weights of liver and kidney, and liver necrosis are observed at dietary concentrations greater than 150 mg/kg fumonisin [13,14,15,16]. However, Li *et al.* [17] reported a decrease in humoral immunity and suppression of lymphocytes in chickens fed 200 mg/kg FB_1_. 

Toxicity of some individual mycotoxins can be enhanced in a synergistic, additive or antagonistic manner when they occur as co-contaminants and are consumed by different animal models [18]. Weibking *et al*. [19] concluded that the effects of AFB_1_ and FB_1_, in chickens and turkeys, when combined, can be more severe than when they are present alone. There is little information on the effects of simultaneous exposure to AFB_1_ and FB_1_ in broilers from commercial strains used in Brazil. The aim of this study was to evaluate changes in total protein concentration and aspartate amino-transferase (AST) activity and liver histopathology in broiler chickens fed diets contaminated with AFB_1_ and/or FB_1_.

## 2. Results and Discussion

Serum concentrations of AST and plasma total protein at day six and 33 post feeding are presented in Table 1. In cases of intoxication causing moderate to severe liver damage, commonly observed changes in liver function tests includes an increase in the serum enzyme AST. At day six post feeding, there was no difference (p > 0.05) in AST levels among treated groups, when compared with the birds from the control group. However, at day 33 post feeding, except for the group that received only 50 mg FB_1_/kg, significant increases (p < 0.05) in serum AST were observed in birds fed all other dietary treatments. The increase in AST levels was highest in groups fed only 200 μg AFB_1_/kg and groups fed 50 μg AFB_1_/kg in combination with FB_1._ These results agree with data reported by Henry *et al.* [14], who fed broilers 80 mg/kg FB_1_ for three weeks and observed an increase in the levels of AST. Weibking *et al*. [19] fed turkey poults a combination of 200 μg AFB_1_/kg and75 mg FB_1_/kg, and also observed an increase in the concentration of AST. Similar results were also observed by Ledoux *et al.* [15] in experiments with both turkeys and broilers.

**Table 1:** Effects of aflatoxin B_1_ (AFB_1_)and fumonisin B_1_ (FB_1_) on serum levels of aspartate amino-transferase (AST) and total protein in broilers ^1^.

AFB_1_	FB_1_	AST (μg/L)		Total Protein (g/dL)	
(μg/kg)	(mg/kg)	6 day ^2^	33 day	6 day	33 day
0	0	19.64^ a^	41.03^ d^	3.485^ a^	3.900 ^b^
0	50	25.03^ a^	65.19^ d^	3.370^ ab^	3.825^ b^
0	200	30.12^ a^	103.50^ b^	3.515^ a^	4.025^ ab^
50	0	26.04^ a^	84.83^ c^	3.485^ a^	3.850^ b^
50	50	25.61^ a^	131.40^ a^	3.350^ ab^	3.925^ b^
50	200	25.32^ a^	141.30^ a^	3.400^ a^	4.000^ ab^
200	0	20.66^ a^	135.20^ a^	3.510^ a^	3.975^ ab^
200	50	24.45^ a^	118.30^ b^	3.200^ b^	3.975^ ab^
200	200	25.61^ a^	118.10^ b^	3.285^ b^	4.100^ a^
SEM		3.85	11.85	0.049	0.052
Source of variation		---------------------------------Probability---------------------------------			
AFB_1_		0.7982	0.0001	0.4381	0.0456
FB_1_		0.3027	0.0043	0.2574	0.0044
AFB_1_ × FB_1_		0.7062	0.0071	0.0001	0.6569

^1^ Results are reported as means of six and four replicates per treatment group in analysis for AST and total protein, respectively.

^2^ Days post feeding.

^a–d ^Values within columns with no common superscript differ significantly (p < 0.05).

**Figure 1:**
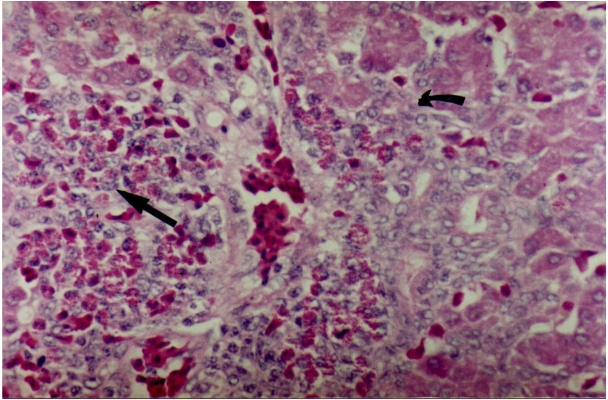
Liver of broilers fed rations containing 200 μg/kg AFB_1_ and 200 mg/kg FB_1_ for 33 days. Note the hyperplasia of bile ducts (curved arrow) and heterophilic infiltration (straight arrow). Haematoxylin and eosin, magnification = 400×.

Accordingly to the increase of AST levels, broilers receiving the highest levels of AFB_1_ and FB_1_ had severe bile duct proliferation and trabecular disorder in liver samples (Figure 1). Groups fed 50 μg AFB_1_/kg alone or in combination with 50 mg/FB_1_/kg showed discrete vacuolar degeneration in the liver, and mild cell proliferation in bile ducts. These changes agree with previous data on the primary effects of AFB_1_ in the liver of broilers [4,9], which constitute an important indicator of intoxication by aflatoxins in the poultry industry. The increase in the levels of serum enzymes is a consequence of hepatocyte degeneration and subsequent leakage of enzymes. Therefore, the significant increase in AST levels may be a very good indicator of toxicity in broilers at levels as low as 50 μg AFB_1_/kg and/or 50 mg FB_1_/kg. An important limitation for using AST as an indicator of liver toxicity is the fact that this enzyme is also present in the heart, skeletal muscle, kidney and brain [20]. In contrast, serum alanine aminotransferase (ALT) activity level is considered the most frequently relied upon laboratory indicator of hepatotoxic effects [20]. However, in our study, no ALT measurements were done, and no skeletal and heart muscles histopathology were performed for confirmation of the exclusive association between the increase in AST levels and liver toxicity observed in the broilers. 

At six days post feeding, there was a significant reduction (p < 0.05) in plasma TP concentration of birds fed diets containing 200 μg AFB_1_/kg alone and in combination with 50 and 200 mg FB_1_/kg diet (Table 1), resulting in a significant AFB_1 _by FB_1_ interaction. At 33 days post feeding, except for the group that were fed a combination of 200 μg AFB_1_/kg + 200 mg FB_1_/kg, and which had higher (p < 0.05) concentration of plasma TP, the TP of birds fed other dietary treatments were not significantly different (p > 0.05) from the control group. These results suggest that birds fed AFB_1_ were able to compensate for the toxic effect on TP as the experiment progressed, returning to normal levels of protein in the plasma after 33 days of continuous feeding of AFB_1_.

According to Tung *et al*. [8], the level of serum protein is considered an important indicator of aflatoxicosis in broilers. However, the data obtained in this study indicate that the reduction in plasma protein levels occurred in the early days of the trial, but was not maintained to the end of the experimental period. One factor that may have contributed to the recovery of normal levels of plasma protein was the low dietary concentrations of AFB_1_ used in this experiment (50 and 200 μg AFB_1_/kg), compared with previous trials. Oguz *et al.* [21] fed broilers 50 and 100 μg AFB_1_/kg diet and also reported no differences in serum total protein at day 33 post feeding. In contrast, Safameher [22] fed broilers 0.5 and 1 mg AFB_1_/kg diet and reported decreased serum total protein concentrations at both days 21 and 42 of age. Similarly, Allameh *et al.* [23] fed broilers 1 mg and 2 mg AFB_1_/kg diet and also reported decreased serum total protein concentrations at both days 21 and 42 of age. Results of the current study and those cited appear to justify the conclusion by Oguz *et al.* [21] that concentrations of AF ≥ 300 μg/kg are required before serum total proteins are affected.

As for other species considered more sensitive to the effects of mycotoxins, Weibking *et al*. [19] fed turkey poults at levels similar to the present study (200 μg AFB_1_/kg and 75 mg FB_1_/kg), and observed a reduction in serum total proteins in the groups fed AFB_1_ alone and in combination with FB_1_ by day 21 of age.

## 3. Experimental Section

The trial was performed in a commercial poultry breeding facility in the city of Descalvado, SP, Brazil. One hundred and eight 1-day-old male broiler chicks, weighing on average 46 g, were used. They came from a 53-week-old commercial broiler lineage (Hybro-PG) and were vaccinated at the hatchery against Marek’s disease. The experimental diets were formulated to meet the nutrient requirements of poultry recommended by National Research Council [24].

Birds were all housed in one cage from day 1 to 7, and received water and a basal diet *ad libitum.* On day 8, birds were randomly assigned to 9 experimental cages with 12 chicks per cage. Individual bird was considered the experimental unit. A completely randomized 3 x 3 factorial design was used, with 3 levels of AFB_1_ (0, 50, and 200 μg AFB_1_) and three levels of FB_1_ (0, 50, and 200 mg FB_1_). Chicks received experimental diets from day 8 to 41. Birds were vaccinated by ocular route against Newcastle disease at 14 d of age using the industrialized (lyophilized) vaccine with the live virus, type B_1_, LaSota strain (NEW VAC-LS^®^).

AFB_1_ and FB_1_ used in the experiment were produced at the Veterinary Medical Diagnostic Laboratory, University of Missouri, Columbia, Missouri, by means of toxigenic strains of *Aspergillus flavus*, and *Fusarium verticilliodes*, according to Ogido *et al.* [18]. The AFB_1_ was resuspended in sterile corn oil, and the suspension was used to produce the experimental diets. The FB_1_ was produced in culture material based on maize, homogenized and sterilized, according to procedures described by Weibking *et al.* [16].

Mixing of the appropriate volumes of the AFB_1 _suspension of and culture material containing FB_1_ with other dietary ingredients, was accomplished in a horizontal/auger mixer (Marconi^®^). Five batches of the diet totaling 550 kg were mixed during the experimental period. Confirmation of the levels of AFB_1_ in the experimental diets was determined by thin-layer chromatography, using the method described by Soares and Rodriguez-Amaya [25]. Procedures described by Shephard *et al.* [26] were used to confirm levels of FB_1 _which were quantified by high performance liquid chromatography (HPLC). Additionally, the basal diet was screened and found to be free of the mycotoxins ochratoxin A and zearalenone. The assay detection limits were 5.0 μg/kg for ochratoxin A and 55.0 μg/kg for zearalenone.

At 14 and 41 days of age (6 and 33 days post feeding, respectively), blood samples were collected by vein puncture (ulnar vein) from 6 birds in each treatment group. The serum was obtained by blood centrifugation in glass tubes for 10 minutes. Serum samples were placed in sterile microtubes and stored in a freezer (-20 ºC) for later analysis of serum AST, using the UV-Kinetic method and a commercial kit LABTEST–Diagnóstica, following the manufacturer's instructions. On days 6 and 33 post feeding, blood samples were also collected from 4 birds from each treatment for determination of plasma total protein. The blood samples were collected in tubes containing penicillin and the anticoagulant ethylenediaminetetraacetic acid (EDTA), at 0.1 ml to 1.0 ml of blood, and total proteins were determined using the Goldberg refractometer (Quimis®). An aliquot of the blood sample was transferred into a capillary tube 7 cm long and 1 mm in diameter, the free end of the tube was closed with flame and the sample centrifuged at 1,200 g, for 5 minutes. The capillary tube was cut at the boundary between the plasma and the globular part and a drop of plasma was placed in a Goldberg refractometer (Quimis®) to be analyzed.

At the end of the trial, six broilers from each treatment group were anesthetized with ether, euthanized by cervical dislocation and necropsied. Liver fragments were collected in 10 per cent neutral buffered formalin. Tissue sections 5 μm thick were stained with haematoxylin and eosin and used for histological evaluation [27].

The data were submitted to factorial analysis (3 × 3), according to the *General Linear Model of SAS*^®^ [28]. Variable means for treatments showing significant differences in the ANOVA were compared using Fisher’s protected least significant difference. All statements of significance are based on the 0.05 level of probability.

## 4. Conclusions

Results of this trial indicated that feeding of AFB_1_ and FB_1_—both alone and in combination—can adversely affect liver function of broiler chickens as characterized by increased serum levels of AST. At the concentrations used in the experiment, only the combination of AFB_1_ and FB_1_ caused a decrease in plasma TP levels. The combination of AFB_1 _and FB_1 _in feeds at these dietary levels indicates a primarily additive effect on serum AST and plasma TP levels of broilers.
